# Potential of New Isolates of *Dunaliella Salina* for Natural β-Carotene Production

**DOI:** 10.3390/biology7010014

**Published:** 2018-02-01

**Authors:** Yanan Xu, Iskander M. Ibrahim, Chiziezi I. Wosu, Ami Ben-Amotz, Patricia J. Harvey

**Affiliations:** 1 Department of Life Science, Faculty of Engineering and Science, University of Greenwich, Kent ME4 4TB, UK; y.xu@greenwich.ac.uk (Y.X.); ibrahii@purdue.edu (I.M.I.); chiziezi.wosu@greenwich.ac.uk (C.I.W.); 2 Nature Beta Technologies (NBT) Ltd., Eilat 88106, Israel; amiba@bezeqint.net

**Keywords:** *Dunaliella salina*, new isolates, characterization, light intensity, β-carotene, carotenoids, correlations, lutein, classification

## Abstract

The halotolerant microalga *Dunaliella salina* has been widely studied for natural β-carotene production. This work shows biochemical characterization of three newly isolated *Dunaliella* *salina* strains, DF15, DF17, and DF40, compared with *D. salina* CCAP 19/30 and *D. salina* UTEX 2538 (also known as *D. bardawil*). Although all three new strains have been genetically characterized as *Dunaliella salina* strains, their ability to accumulate carotenoids and their capacity for photoprotection against high light stress are different. DF15 and UTEX 2538 reveal great potential for producing a large amount of β-carotene and maintained a high rate of photosynthesis under light of high intensity; however, DF17, DF40, and CCAP 19/30 showed increasing photoinhibition with increasing light intensity, and reduced contents of carotenoids, in particular β-carotene, suggesting that the capacity of photoprotection is dependent on the cellular content of carotenoids, in particular β-carotene. Strong positive correlations were found between the cellular content of *all-trans* β-carotene, 9-*cis* β-carotene, *all-trans* α-carotene and zeaxanthin but not lutein in the *D. salina* strains. Lutein was strongly correlated with respiration in photosynthetic cells and strongly related to photosynthesis, chlorophyll and respiration, suggesting an important and not hitherto identified role for lutein in coordinated control of the cellular functions of photosynthesis and respiration in response to changes in light conditions, which is broadly conserved in *Dunaliella* strains. Statistical analysis based on biochemical data revealed a different grouping strategy from the genetic classification of the strains. The significance of these data for strain selection for commercial carotenoid production is discussed.

## 1. Introduction

Natural carotenoids have gained increasing attention in recent years because of their health benefits compared to synthetic carotenoids [1]. These lipophilic compounds comprise a range of carotenes and xanthophylls, and their health benefits generally derive from their ability to quench oxygen radicals and absorb potential damaging visible light [2,3,4]. Carotenoids predominantly occur in their *trans* configuration but are also naturally found in their *cis* configuration [5]. Methods for producing synthetic carotenoids especially β-carotene and zeaxanthin are well-established [1], however synthetic carotenoids are predominantly *all-trans* compounds and are of questionable benefit [6]. By contrast, intake of food supplements enriched with natural β-carotene containing both *cis-* and *trans-* stereoisomers is linked with mitigation of a range of diseases including atherosclerosis, diabetes, psoriasis and ophthalmologic diseases [7,8,9]. *9-cis* β-carotene is of particular nutritional and medical interest as a retinoid precursor and is associated with therapeutic effects in a number of diseases as well as possessing a good adverse effect profile [10]. This stereoisomer is difficult to synthesize chemically, it is not produced biologically by heterotrophs such as bacteria or yeasts, through fermentation, and it is present in only low amounts in fruits and vegetables [11,12]. 9*-cis* β-carotene, along with the *13-cis* and *15-cis* isomers found in food and naturally-occurring substances, may serve an important function in human physiology that cannot be replaced by synthetic β-carotene.

Microalgae are considered the richest sources of natural carotenoids, especially strains of the Chlorophyta such as *Dunaliella salina*, *Haematococcus pluvialis*, and various *Chlorella* species [3]. The content of lutein in marigold flowers for example is commonly reported to be 0.3 mg·g^−1^, but in microalgae, the content can be over 4 mg·g^−1^ [13]. *Dunaliella* strains are well known for being rich in lutein, zeaxanthin and β-carotene [14] and *D. salina* has been particularly widely studied as it is the richest source of natural β-carotene [15] and contains high content of the *9-cis* isomer (~50% of the total β-carotene) [11,12]. Other valuable carotenoids with potential medical value are also present in *D. salina* including violaxanthin, antheraxanthin, zeaxanthin, α-carotene, and lycopene [16]. The genus *Dunaliella* contains a number of species and many strains which have been identified under the same species possess various carotenogenic abilities and carotenoid compositions [17].

In this study, as part of the D-Factory, an European Union (EU) funded project, three new strains of *Dunaliella* species, DF15, DF17, and DF40 isolated from salt ponds in Israel and Spain were characterized. The strains have been genetically identified as strains of *Dunaliella salina* but under different subgroups (Marine Biological Association—MBA, www.mba.ac.uk/culture-collection/). The biochemical properties of the strains were examined in this study in comparison to the known carotene hyperaccumulator, *D. salina* UTEX 2538, also classified as *D. bardawil* in some studies [4] and *D. salina* CCAP19/30, which has been found to be very similar to a *D. tertiolecta* strain and does not accumulate β-carotene under stress [18], in order to assess their potential for the commercial production of carotenoids and provide further insight into carotenoid metabolism.

## 2. Materials and Methods

### 2.1. Algal Strains and Cultivation

*D. salina* UTEX 2538 was obtained from the Culture Collection of Algae at The University of Texas at Austin (UTEX, Austin, TX, USA) and *D. salina* CCAP 19/30 was obtained from the Culture Collection of Algae and Protozoa at Scottish Marine Institute (CCAP, Scotland, UK). D-Factory strains DF15 and DF17 were isolated from a salt pond in Eilat, Israel, and DF40 was isolated from a salt pond in Monzon, Spain. The new isolates were identified as strains of or closely related to *Dunaliella salina (bardawil)* by The Marine Biological Association (MBA, Plymouth, Devon, UK) and are now deposited at the MBA culture collection (www.mba.ac.uk/culture-collection/). Algae were cultured in Modified Johnsons Medium [19] containing 10 mM NaHCO_3_ with the pH value adjusted to 7.5 with 10 mM Tris-buffer, and 1.5 M NaCl, which has been tested as the optimal salinity for cell growth of the strains. Cultures were maintained in a temperature-controlled growth chamber at 20 ± 2 °C with illumination provided under a 12 h light, 12 h dark cycle (12/12 LD) by white light-emitting diode (LED) lights with a light intensity of ~200 µmol photons·m^−2^·s^−1^.

For algal cultivation, small stock cultures were grown to mid-log phase at 25 °C in an incubator and diluted 1 in 50 (*v*/*v*) as inoculum for larger cultures in each experiment. Erlenmeyer flasks containing 500 mL culture each were maintained at 25 °C in an ALGEM Environmental Modeling Labscale Photobioreactor (Algenuity, Bedfordshire, UK) with strictly controlled conditions of light, temperature and mixing level. Under 12/12 LD conditions, cell growth under a range of light intensities (200, 500, 1000, and 1500 μmol·m^−2^·s^−1^) of white LED light were compared. Each growth condition was set up at least in triplicate. Cell growth was monitored automatically in the bioreactor by recording the value obtained for light scatter at 725 nm in optical density (OD) units. Cultures were mixed at 100 rpm for 10 min every hour before measuring the OD. Cell concentration was determined by counting the cell number in culture broth using a haemocytometer after fixing the cells with 2% formalin. The maximum specific growth rate of all cultures was calculated to compare cell growth under different conditions.

### 2.2. Microscopy Observations

The Eclipse Ti-U inverted research microscope (Nikon, Tokyo, Japan) with a Nikon Digital Sight DS-Fi1 camera system was used to take brightfield microscopy photographs of cells of each *Dunaliella* strain. The objective lens used was Nikon Splan Fluor ELWD 60×/0.7 and the ocular lens was Nikon CFI 10×/22. The NIS-Elements Advanced Research Microscope Imaging Software (Nikon, Tokyo, Japan) was used to acquire the photos. Differential interference contrast (DIC) microscopy photographs were also obtained using a confocal microscope system ZEISS LSM 880 (Carl Zeiss Microscopy GmbH, Jena, Germany). The ZEISS Plan Apochromat 63×/1.4 oil DIC objective lens and the Carl Zeiss PI 10×/23 ocular lens were used. Images were acquired and analyzed through the ZEN 2.1 LSM software (Carl Zeiss Microscopy GmbH).

### 2.3. Algal Biomass Analysis

Algae grown under different light intensities were harvested during mid log phase of growth at the end of the light period. Pigments were extracted from the biomass harvested from 1 mL samples of the cultures using 1 mL of 80% (*v*/*v*) acetone. The absorbance of the acetone extract after clarification at the centrifuge was measured at 480 nm for total carotenoids using an ultraviolet (UV)/Vis spectrophotometer. The content of total carotenoids was calculated according to Strickland & Parsons [20]. Chlorophyll a, b, and total chlorophyll were evaluated by measuring absorbance of the acetone extract at 664 nm and 647 nm and calculated according to Porra et al. [21].

The compositions of pigments extracted from different strains were analyzed using high performance liquid chromatography (HPLC) with diode array detection (DAD) (Agilent Technologies 1200 series, Agilent, Santa Clara, CA, USA). Carotenoid standards of *all-trans* α-carotene, *all-trans* β-carotene and zeaxanthin were obtained from Sigma-Aldrich, Inc. (Merck KGaA, Darmstadt, Germany). Lutein and *9-cis* β-carotene were obtained from Dynamic Extractions (Tredegar, UK). Carotenoids and chlorophylls were extracted from freshly harvested cells using methyl tert-butyl ether (MTBE) and Methanol (MeOH) (20:80) as extraction solvent. 15 mL of algal culture was centrifuged at 3000 *g* at 18 °C for 5 min and the pellet was extracted with 10 mL MTBE-MeOH (20:80) and sonicated for 20 s. The sample was clarified by centrifugation at 3000 *g* at 18 °C for 5 min, then 1–2 mL of the supernatant was filtered through 0.45 µm syringe filter into amber HPLC vials. It was then analyzed using a YMC30 250 × 4.9 mm I.D S-5µ HPLC column with DAD at 25 °C, and isocratic elution with 80% methanol: 20% MTBE, flow rate of 1 mL·min^−1^, pressure of 90 bar. The quantities of *9-cis* and *all-trans* β-carotene, *all-trans* α-carotene, lutein, and zeaxanthin in the biomass were determined from the corresponding standard curves. Glycerol, known to be regulated by salinity, was determined according to the procedures described in a Xu et al. [18].

### 2.4. Oxygen Evolution and Dark Respiration

Cells were harvested during the exponential phase and NaHCO_3_ was added to a final concentration of 10 mM 5 min before the start of each measurement. The rates of net O_2_ evolution and dark respiration were measured as described by Brindley et al. [22] at 25 °C using a Clark-type electrode (Hansatech Instruments Ltd, Norfolk, UK) [23]. O_2_ evolution was induced with 1500 μmol photons·m^−2^·s^−1^ actinic light. After initial 30 min of dark adaption, O_2_ evolution was measured for 5 min followed by dark respiration for 20 min. The average net rate of photosynthesis was then determined from the oxygen concentration gradient recorded over 5 min, dO_2_/dt. Dark respiration was determined by following the same procedure, except that oxygen uptake was calculated from data recorded during the last 5 min of the 20 min experiment. Sodium dithionite was used to calibrate the oxygen electrode. 

### 2.5. Statistical Analysis

The data generated in this study was analyzed in R (Rstudio, Boston, MA, USA). A two-way analysis of variance (ANOVA) analysis was performed to study the relationships of a series of variables measured with two factors in this work: strain and light intensity. The two-way ANOVA tests three omnibus effects: the main effect of strain or light intensity, and the interaction effect between these two factors. Correlation analysis was used to evaluate the association between each pair of the variables and the Pearson correlation method was chosen to measure the linear dependence between two variables. In correlation analysis, a correlation coefficient (the Pearson Product Moment correlation coefficient) was estimated for each pair of the variables studied. Whether or not an observed correlation is statistically significant or not was evaluated by *P* values (significant when *P* ≤ 0.05). Hierarchical cluster analysis is based on the strength of the correlations and the distance in the clustering dendrogram reflects the dissimilarity among these parameters. Traits examined with strong correlations are grouped as a cluster. A principle component analysis was carried out using the whole data set to reveal the relatedness between the examined traits.

## 3. Results

### 3.1. Cell Growth

**Figure 1:**
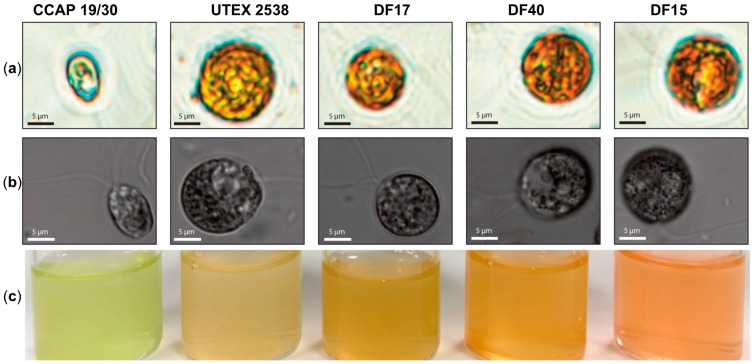
Microscopy observation of *Dunaliella* cells and photographs of stationary phase cultures of CCAP 19/30, UTEX 2538, DF17, DF40 and DF15 grown under a light intensity of 100~200 µmol·m^−2^·s^−1^ at 20 °C. (**a**) Microscopy photographs taken through a light microscope (Nikon Eclipse Ti-U) with a magnification of 600×; (**b**) Differential interference contrast (DIC) microscopy photographs taken through a confocal microscope (ZEISS LSM 880) with a magnification of 630×. (**c**) Photographs of the cultures obtained for each *Dunaliella* strain grown under identical conditions.

The work presented here shows biochemical and biophysical characterization of the three newly isolated *D. salina* strains: DF15, DF17 and DF40 compared with *D. tertiolecta* CCAP 19/30 and *D. salina (bardawil)* UTEX 2538, cultured under a series of light intensities. Cultures of five *Dunaliella* strains: CCAP 19/30, DF15, DF17, DF40, and UTEX 2538, were each maintained under identical conditions of light of 100~200 µmol·m^−2^·s^−1^ in the incubator until stationary phase, and cells were photographed using a light microscope and a confocal microscope. The five strains differed in cell shape and cell size from each other (Figure 1a,b), and the cultures of each strain were differently colored (Figure 1c). Cells of CCAP 19/30 maintained oval shapes and a green color throughout, while the four *D. salina* strains gradually changed from oval to round shapes and from green to orange during cultivation. An estimation of the average cell sizes based on at least 100 cells observed under the microscope showed that DF15 has a similar cell size to UTEX 2538 (~2000 µm^3^); DF40 and DF17 have slightly smaller sizes (~1200 and 1000 µm^3^ respectively), and CCAP 19/30 has a significantly smaller cell size than all strains examined (~200 µm^3^).

**Figure 2:**
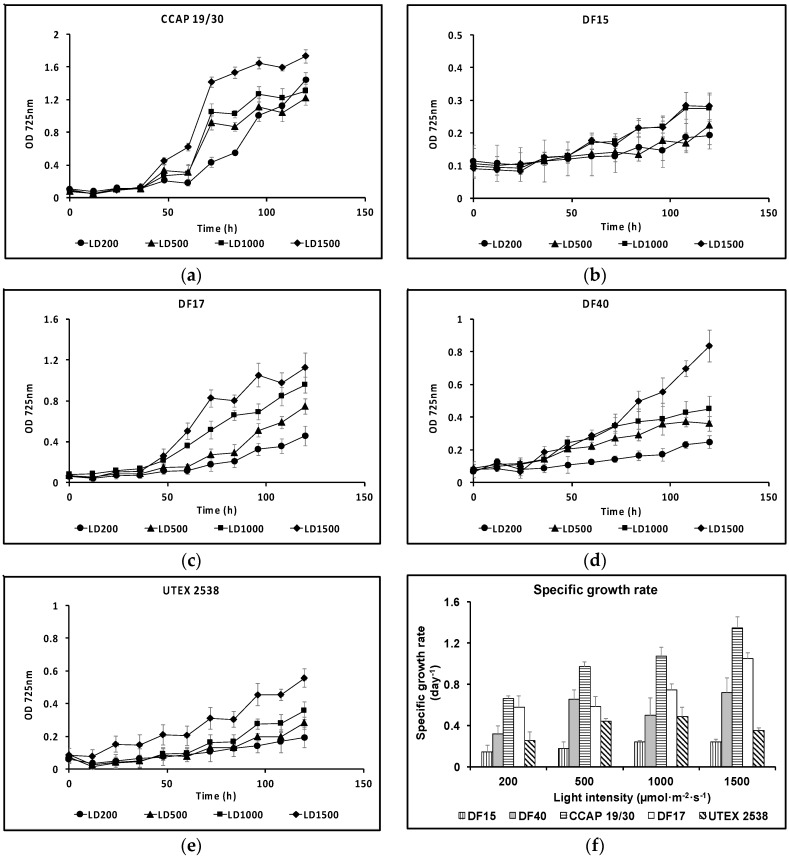
Growth curves for the five *Dunaliella* strains: (**a**) CCAP 19/30; (**b**) DF15; (**c**) DF17; (**d**) DF40; (**e**) UTEX 2538 each grown under four identical light intensities of 200, 500, 1000 and 1500 µmol·m^−2^·s^−1^ at a light/dark cycle of 12 h light and 12 h dark (LD200, LD500, LD1000 and LD1500); (**f**) specific growth rates of each strain grown under the four light intensities. Each culture condition was set up in triplicate.

Growth curves for the five strains cultivated under the same conditions of different light intensities of 200, 500, 1000, and 1500 µmol·m^−2^·s^−1^ are shown in Figure 2, from which the maximum specific growth rate was calculated for each growth condition. Generally, these strains grew at a faster rate under higher light intensities. This is clearly shown for CCAP 19/30 and DF17. All strains showed the slowest growth rates under 200 µmol·m^−2^·s^−1^ light intensity. In DF15 and UTEX 2538, when increasing the light intensity from 1000 µmol·m^−2^·s^−1^ to 1500 µmol·m^−2^·s^−1^, no further improvement in cell growth rate was observed. It is likely that the optimal light intensity for fastest growth of DF15 or UTEX 2538 is around 1000 µmol·m^−2^·s^−1^, while 1500 µmol·m^−2^·s^−1^ or higher is optimal for the other three strains under the specific growth conditions used (white LED lights under 25 °C with 10 min mixing at 100 rpm every hour). DF15 had the slowest growth rate and CCAP 19/30 the fastest.

### 3.2. Photosynthesis and Respiration

**Figure 3:**
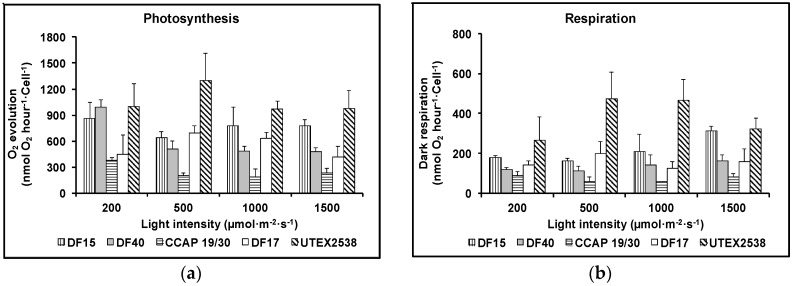
Photosynthesis (**a**) and respiration (**b**) of the five *Dunaliella* strains cultivated under four light intensities of 200, 500, 1000, and 1500 µmol·m^−2^·s^−1^. Samples were taken at the mid log phase and all culture conditions were repeated at least in triplicates.

Figure 3a shows that as the light intensity increased, the rate of photosynthesis decreased for DF17, DF40 and CCAP 19/30, indicating that these three strains are susceptible to photoinhibition. However, DF15 and UTEX 2538 did not exhibit photoinhibition with increase in light intensity, suggesting that these two strains have a more robust photoprotection mechanism. Figure 3b shows that the dark respiration rate patterns were similar for DF17, DF40, and CCAP 19/30. These three strains showed a slight decrease or no change in dark respiration rate with the increase in light intensity. DF15 and UTEX 2538 had a similar pattern to each other and their respiration rate increased slightly with increase in light intensity. From statistical analysis using two-way ANOVA, both strain difference and light intensity were significant factors affecting photosynthesis; less significant was the interaction between them. However, light intensity showed no significant impact on dark respiration, but strain played a major role in the observed differences in dark respiration (Table 1).

### 3.3. Pigment Composition

**Figure 4:**
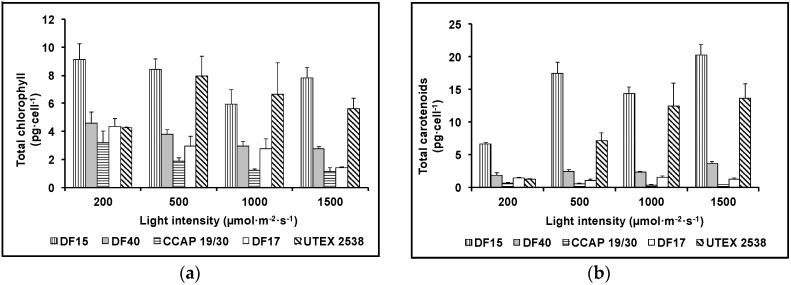
Cellular content of total chlorophyll (**a**) and total carotenoids (**b**) of the five *Dunaliella* strains grown under four light intensities of 200, 500, 1000, and 1500 µmol·m^−2^·s^−1^. Samples were taken at the mid log phase and all culture conditions were repeated at least in triplicates.

Cellular contents of total chlorophyll and total carotenoids were determined for the five *Dunaliella* strains grown under the four light intensities (200, 500, 1000, and 1500 µmol·m^−2^·s^−1^) using UV/Vis spectrometry (Figure 4). Generally, the cellular content of total chlorophyll decreased while total carotenoids increased with the increase in light intensity for all five *Dunaliella* strains. Statistical analysis showed that strain difference significantly affected total carotenoids and total chlorophyll content, although total carotenoids and total chlorophyll content also responded significantly to light intensity (Table 1).

**Figure 5:**
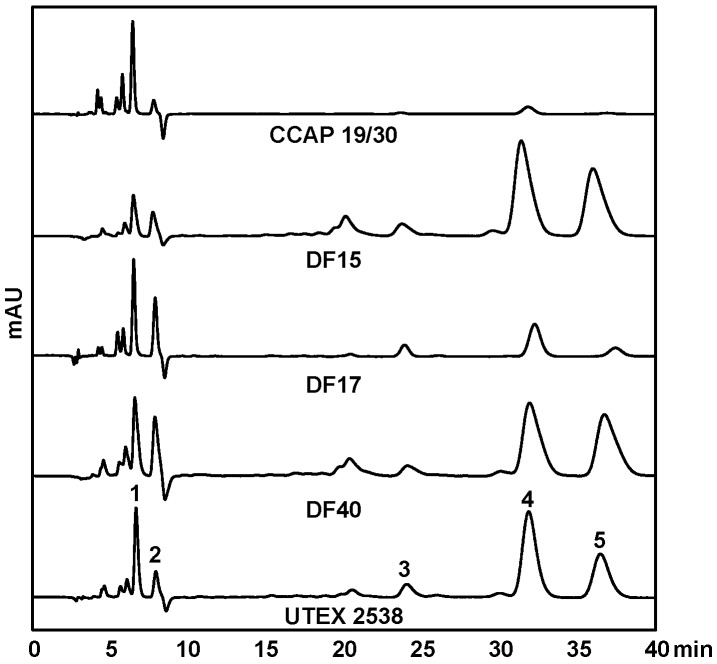
HPLC chromatograms of MTBE/ethanol extracts of the five *Dunaliella* strains cultivated under 1500 µmol·m^−2^·s^−1^. The major peaks shown are: (1) lutein, (2) zeaxanthin, (3) *all-trans* α-carotene, (4) *all-trans* β-carotene and (5) *9-cis* β-carotene.

HPLC-DAD was used to quantify the contents of major carotenoids, namely lutein, zeaxanthin, *all-trans* β-carotene, *9-cis* β-carotene, and *all-trans* α-carotene, in each strain acclimated in response to four light intensities, to understand the effect of light in carotenoid metabolism. Figure 5 shows HPLC chromatograms of the pigment extracts from the five *Dunaliella* strains grown under the light intensity of 1500 µmol·m^−2^·s^−1^. It is clear that CCAP 19/30 does not accumulate β-carotene even under high light intensity. DF15, DF40 and UTEX 2538 have a similar pigment profile and β-carotene dominates the carotenoid composition. DF17 produced a higher relative amount of zeaxanthin under high light stress compared with the other strains, indicating the important role of zeaxanthin in DF17 for photoprotection.

**Figure 6:**
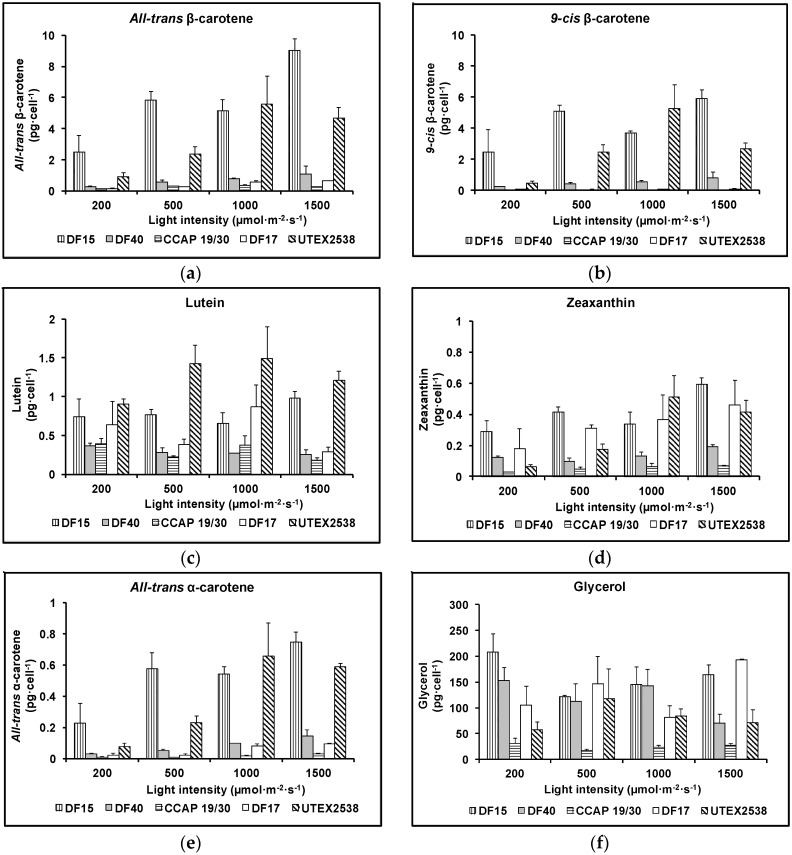
Cellular contents of (**a**) *all-trans* β-carotene, (**b**) *9-cis* β-carotene, (**c**) lutein, (**d**) zeaxanthin, (**e**) *all-trans* α-carotene and (**f**) glycerol in the five *Dunaliella* strains cultivated under four light intensities of 200, 500, 1000, and 1500 µmol·m^−2^·s^−1^. Samples were taken at the mid log phase and all culture conditions were repeated at least in triplicate.

The major difference between the strains was their ability to accumulate β-carotene. As shown in Figure 6a,b, the contents of *all-trans* and *9-cis* β-carotene increased with increasing light intensity in all five strains apart from UTEX 2538, which produced the highest cellular amount of *all-trans* (5.6 ± 1.8 pg·cell^−1^) or *9-cis* β-carotene (5.3 ± 1.5 pg·cell^−1^) under 1000 µmol·m^−2^·s^−1^. Under the highest light intensity studied (1500 µmol·m^−2^·s^−1^), the cellular contents of *all-trans* β-carotene and *9-cis* β-carotene were 9.0 ± 0.7 and 5.9 ± 0.6 pg·cell^−1^ in DF15; 1.1 ± 0.5 and 0.8 ± 0.4 pg·cell^−1^ in DF40; and 0.6 ± 0.0 and 0.1 ± 0.0 pg·cell^−1^ in DF17. In CCAP 19/30, the highest *all-trans* β-carotene content (0.3 ± 0.0 pg·cell^−1^) was obtained at 1000 µmol·m^−2^·s^−1^, and only a very small amount of *9-cis* β-carotene was detected at all light intensities (~0.01 pg·cell^−1^). All five strains achieved the highest *all-trans* β-carotene productivity at 1500 µmol·m^−2^·s^−1^ (3.2 ± 0.0, 3.5 ± 0.0, 1.3 ± 0.0, 2.6 ± 0.0 and 2.9 ± 0.0 mg·L^−1^·day^−1^ for CCAP 19/30, DF15, DF17, DF40 and UTEX 2538 respectively), and also the highest *9-cis* β-carotene productivity at 1500 µmol·m^−2^·s^−1^ except that UTEX 2538 has the highest yield of *9-cis* β-carotene at 1000 µmol·m^−2^·s^−1^ (0.2 ± 0.0, 2.3 ± 0.0, 0.2 ± 0.0, 2.0 ± 0.0 and 2.2 ± 0.0 mg·L^−1^·day^−1^ for CCAP 19/30, DF15, DF17, DF40 and UTEX 2538 respectively). From the two-way ANOVA analysis, the cellular contents of *all-trans* or *9-cis* β-carotene were found to vary significantly among strains and under different light intensities (Table 1). CCAP 19/30, DF17 and DF40 had similar responses to increasing light with a mild β-carotene accumulation, while DF15 and UTEX 2538 significantly increased β-carotene content with increasing light (Figure 6a,b). DF15 and UTEX 2538 have significantly higher cellular contents of *all-trans-* or *9-cis* β-carotene than the other three strains and DF15 contains a higher cellular content of β-carotene than UTEX 2538 under most of the light conditions. UTEX 2538, already known to be a massive carotene-accumulating strain [4], had faster growth rates than DF15 under all light intensities examined, as shown in Figure 2f. On the other hand, DF15 accumulated a high carotene content even under the lowest light intensity tested here. In *Dunaliella*, variation in β-carotene content has been reported to correlate with the integral irradiance received during a division cycle and to be a specific mechanism of photoprotection [24], which may explain why DF15 has a higher cellular content of β-carotene than UTEX 2538. DF15 has the advantage of accumulating a large amount of β-carotene even without light stress (Figure 6), and also highest productivity of both *all-trans* and *9-cis* β-carotene under light stress, therefore has great potential for the commercial production of β-carotene with less light energy input required.

The cellular content of lutein in the five *Dunaliella* strains grown under various light intensities is shown in Figure 6c. All strains accumulated considerably different amounts of lutein and the response to increasing light intensities varied among different strains. Lutein increased with light intensity from 200 µmol·m^−2^·s^−1^ to 1000 µmol·m^−2^·s^−1^ and then decreased when light increased to 1500 µmol·m^−2^·s^−1^ in UTEX 2538. In DF15, lutein content did not change with light intensity from 200 µmol·m^−2^·s^−1^ to 1000 µmol·m^−2^·s^−1^ and only increased from 1000 to 1500 µmol·m^−2^·s^−1^. Both DF15 and UTEX 2538 accumulated significantly larger amounts of lutein under high light compared with the other strains. DF17 had the highest lutein content at 1000 µmol·m^−2^·s^−1^, and the lowest at 1500 µmol·m^−2^·s^−1^. Two-way ANOVA shows the cellular content of lutein is significantly affected by both the strain and light intensity.

Figure 6d shows that zeaxanthin content in all strains increased with light intensity. DF15 accumulated the highest amount of zeaxanthin, followed by DF17, UTEX 2538, DF40 and CCAP 19/30 accumulated the lowest amount. Two-way ANOVA analysis (Table 1) shows that the factors of strain and light intensity determined the accumulation of zeaxanthin. Zeaxanthin accumulation was significantly different among strains and at different light intensities. Among the different strains, DF17 and UTEX 2538 had similar responses in terms of zeaxanthin accumulation.

The cellular content of *all-trans* α-carotene of the five strains grown under different light intensities is shown in Figure 6e and the cellular content of glycerol is shown in Figure 6f. The content *of all-trans* α-carotene in DF15 or UTEX 2538 was much higher than that in the other three Strains. α-carotene is the precursor of lutein but surprisingly α-carotene did not respond to light stress in the same way as lutein. *All-trans* α-carotene increased with the light intensity in all strains examined, and its response to increasing light intensity was very similar to the pattern of accumulation obtained for *all-trans* and *9-cis* β-carotene. 

### 3.4. Statistical Analysis

Whilst the accumulated data permit elucidation of strain differences for carotenoid production, they also provided the opportunity to explore the use of statistical analysis to provide new insights into carotenoid metabolism coupled to the interdependent metabolic functions of photosynthesis and respiration. This was possible with the large set of data generated across five strains and four light intensities combined with tools of ANOVA analysis, correlation analysis, and principal component analysis used in this study. With the quantitative data obtained for the five *Dunaliella* strains, statistical analysis was used as a tool in order to assess the strength of the correlations among the carotenoids and other cell growth parameters and examine the differences among the five strains. A correlation and clustering analysis was performed on the growth, photosynthesis and pigment data presented, to all five strains grown under four light conditions. The analysis was performed for each strain using all variables examined in this study (*all-trans* β-carotene, *9-cis* β-carotene, glycerol, lutein, zeaxanthin, *all-trans* α-carotene, photosynthesis, respiration, total carotenoids, total chlorophyll, and specific growth rate). Among them, glycerol is known to maintain osmotic balance in *Dunaliella* strains and as expected, the cellular content of glycerol would not respond to changes in light intensity, as shown in Figure 6f. Glycerol content therefore was used to index the analysis.

**Figure 7:**
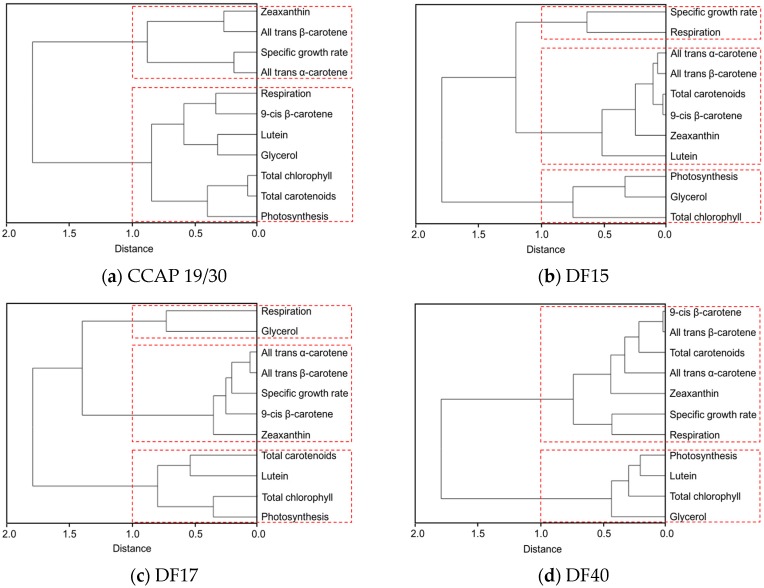

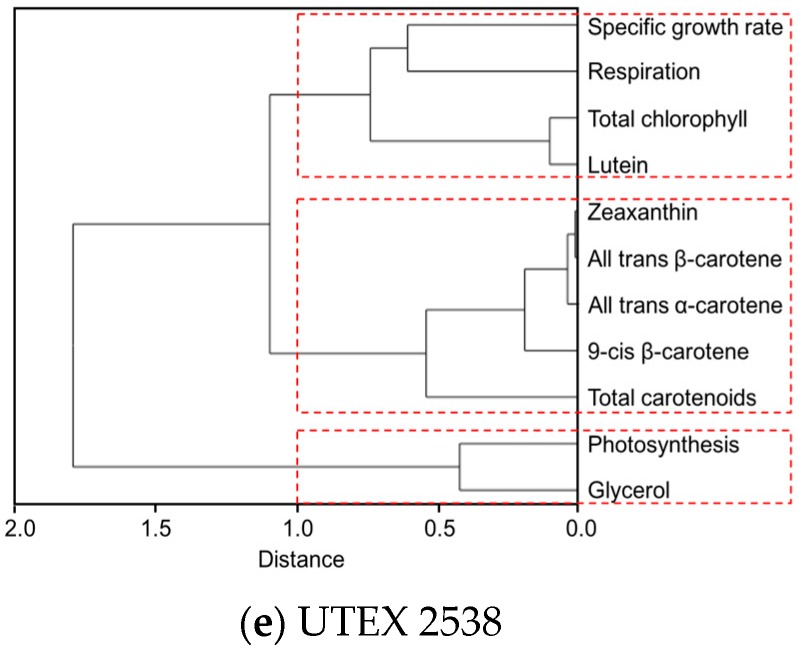
Cluster dendrograms of *all-trans* β-carotene, *9-cis* β-carotene, glycerol, lutein, zeaxanthin, *all-trans* α-carotene, photosynthesis, respiration, total carotenoids, and total chlorophyll for all five *Dunaliella* strains cultivated at four light intensities. (**a**) CCAP 19/30; (**b**) DF15; (**c**) DF17; (**d**) DF40 and (**e**) UTEX 2538.

**Figure 8:**
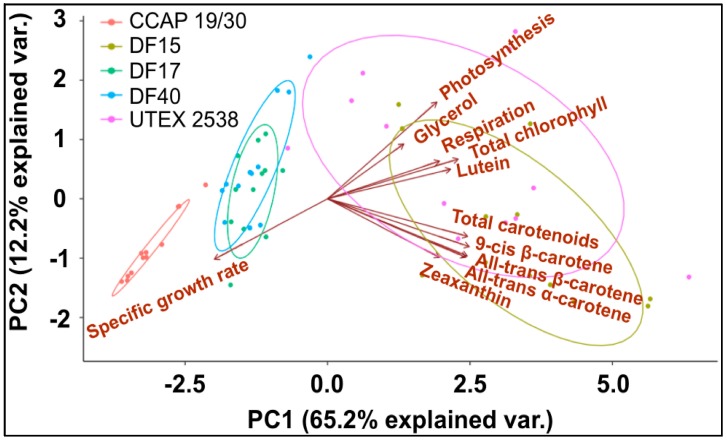
Principle component analysis of 11 traits (*all-trans* β-carotene, *9-cis* β-carotene, glycerol, lutein, zeaxanthin, *all-trans* α-carotene, photosynthesis, respiration, total carotenoids, total chlorophyll, and specific growth rate) for all five *Dunaliella* strains cultivated at four light intensities.

The clustering dendrogram of the examined traits for each strain is shown in Figure 7 and depicts graphically several features of note amongst the strains. First, it shows that the individual carotenoids of *all-trans* β-carotene, *9-cis* β-carotene, zeaxanthin and *all-trans* α-carotene in the four *D. salina* strains are strongly correlated with each other but significantly not with lutein, except in CCAP 19/30. From this, it is clear that there is greater similarity between the four *D. salina* strains (DF15, DF17, DF40, and UTEX 2538) than with the CCAP19/30 strain. Second, the correlation analysis shows that accumulation of carotenoids is positively correlated with photosynthesis over all light intensities for the *D. salina* strains (also shown in Figure 8), signifying a role for carotenoids in photoprotection. Third, lutein is not correlated closely with the other carotenoids, but correlates more strongly with photosynthesis and respiration. This result suggests an important and not hitherto identified role for lutein in coordinated control of the cellular functions of photosynthesis and respiration in response to changes in light conditions, which is moreover broadly conserved in *Dunaliella* strains. Glycerol, which was not expected to change with light intensity, is weakly correlation with the different carotenoids in the *Dunaliella* strains as anticipated, but also correlates more closely with either photosynthesis or respiration.

A principle component analysis was performed with all strains growing at all tested conditions as shown in Figure 8. The examined 11 traits can be roughly grouped into four groups as shown in the graph, where *all-trans* β-carotene, *all-trans* α-carotene, *9-cis* β-carotene and zeaxanthin were clustered closely, lutein, respiration and total chlorophyll were found in a second cluster, glycerol and photosynthesis were closely correlated, and the specific growth rate stands separately. The formation of two separate clusters of the carotenoids indicates two functionally distinct mechanisms for coordinated adaptation to changes in light conditions, broadly conserved between DF15, DF40, CCAP 19/30, DF17 and UTEX 2538. More importantly, it shows that DF17 and DF40 performed similarly under the tested environmental conditions; that DF15 is closely related to UTEX 2538, and that CCAP 19/30 is different compared to all the other strains.

## 4. Discussion

In photosynthesis, light energy absorbed by the chlorophyll- and carotenoid-binding complexes of photosystem II is transferred to reaction centers to drive photochemistry. Excess light energy will cause light-induced damage of photosynthetic apparatus or photo-oxidative damage. Photosynthetic organisms have evolved a robust repair mechanism to replace the photodamaged photosystems; however, when the rate of photodamage exceeds the repair cycle, photosynthetic efficiency will be impaired [25]. Based on this study, it is apparent that CCAP 19/30, DF17 and DF40 are susceptible to photoinhibition, while photosynthetic efficiency of DF15 and UTEX 2538 was not affected by high light and was maintained high over all light intensities studied, suggesting they have developed better photoprotective mechanisms against light stress. In [18], CCAP 19/30 was shown to increase the intracellular glycerol content with increasing light intensity above 500 µmol photons m^−2^·s^−1^, but not carotenoids, as is also shown here (see Figure 6f). Glycerol was proposed to have multiple functions to protect and maintain growth of CCAP 19/30 cells not only in conditions of high salinity but also under high light intensities by stabilizing the photosynthetic apparatus for maximum performance, a role normally attributed to carotenoids [18]. In the present work, all cultures were grown at the same salinity, and the statistical analysis for all strains examined together showed no significant changes in glycerol with light intensity. Nevertheless, it is possible that the findings reported in [18] refer more widely to all strains when compared under constant salinity.

Carotenoids are variously involved in harvesting light for photosynthesis as well as preventing photoinhibition under high light stress. Exposure to white light is associated with generation of reactive oxygen species (ROS), which have been shown to replace light in the induction of hyper-accumulation of carotenoids [26]. β-carotene is also associated with photoprotection and most of the beneficial effects of β-carotene is attributed to its ability to prevent oxidation processes by quenching singlet oxygen (^1^O_2_*) once formed or terminating free radical chain reactions as a result of the presence of the polyene chain, with *9-cis* β-carotene being a better scavenger of free radicals than *all-trans* β-carotene [27]. DF15 and UTEX 2538, which showed no evidence of photoinhibition with increase in light intensity, also accumulated large amounts of carotenoids, especially β-carotene, compared to the other strains.

The fact that DF15 and UTEX 2538 accumulated very large amounts of β-carotene over all light intensities is noteworthy. The accumulation of β-carotene in *D. salina* when exposed to high light mainly occurs in the β-carotene plastoglobuli, while the thylakoidal β-carotene content remains relatively unchanged [28,29]. These plastoglobuli have also been shown to contain many enzymes found in the eyespot of other flagellate algae [30,31]. However, most of the proteins that are required for the eyespot function are no longer found in *Dunaliella* and no eyespot structural elements could be found in *Dunaliella* [32]. This suggests that the plastoglobuli were once components of a functional eyespot of *Dunaliella*. The β-carotene in the eyespot probably played a crucial role in perception of light, but once it lost its function, the non-functional eyespot acted as a β-carotene storage compartment. It is possible therefore that both DF15 and UTEX 2538 accumulated very large amounts of β-carotene in a vestigial eye-spot.

Zeaxanthin is linked to energy dissipation when excess light is absorbed via the xanthophyll cycle [33,34]. Zeaxanthin receives excess excitation energy from excited-state singlet chlorophyll (^1^Chl^*^) and dissipates it harmlessly and rapidly as heat in a process that is commonly assessed as non-photochemical quenching (NPQ) of chlorophyll fluorescence [33,34]. The carotenoids participating in this cycle are the only carotenoids present in the photosynthetic membrane that undergo very rapid, light-triggered concentration changes. High light induces de-epoxidation of violaxanthin and converts it into zeaxanthin, leading to its accumulation. This process is reversed in low light conditions. The accumulation of zeaxanthin in *Dunaliella* has been shown to parallel the accumulation of photodamaged Photosystem II (PSII) centers in the chloroplast thylakoids and decays with chloroplast recovery from photoinhibition [35]. In the present work, the increase in zeaxanthin content in high light, therefore shows that these strains have an efficient photoprotective mechanism also based on the xanthophyll cycle.

Statistical analysis tools used here have been able to reveal the correlative relationships between different carotenoids (lutein, zeaxanthin, *all-trans* and *9-cis* β-carotene and α-carotene) and the relationships between carotenoids and photosynthesis and respiration. In particular, they have identified a strong positive correlation of lutein with photosynthesis and respiration (Figure 7 and Figure 8). In humans, lutein influences brain function through a variety of mechanisms that are not well understood, but its accumulation in brain mitochondria has been proposed to protect these organelles from oxidative damage [36]. Lutein also specifically accumulates in the retina of the eye and has been linked with protection against mitochondrial stress and with mitochondrial biogenesis [37]. In plants there is a close interdependence between respiration and photosynthesis for the flow of adenosine triphosphate (ATP), nicotinamide adenine dinucleotide phosphate (NAD(P)H) and carbon skeletons such that excess photosynthetic reducing equivalents formed by photosynthesis in light can be removed in mitochondrial respiration to reduce the tendency for reactive oxygen species (ROS) accumulation and photoinhibition [38] and thereby regulate the NAD(P)H: oxygen ratio to avoid cell death [39]. The clustering of lutein, photosynthesis, chlorophyll and respiration reported here attests to the strong interdependence between respiration and photosynthesis to regulate the redox state of the cell [38], and in *Dunaliella* both are linked to lutein accumulation.

**Figure 9:**
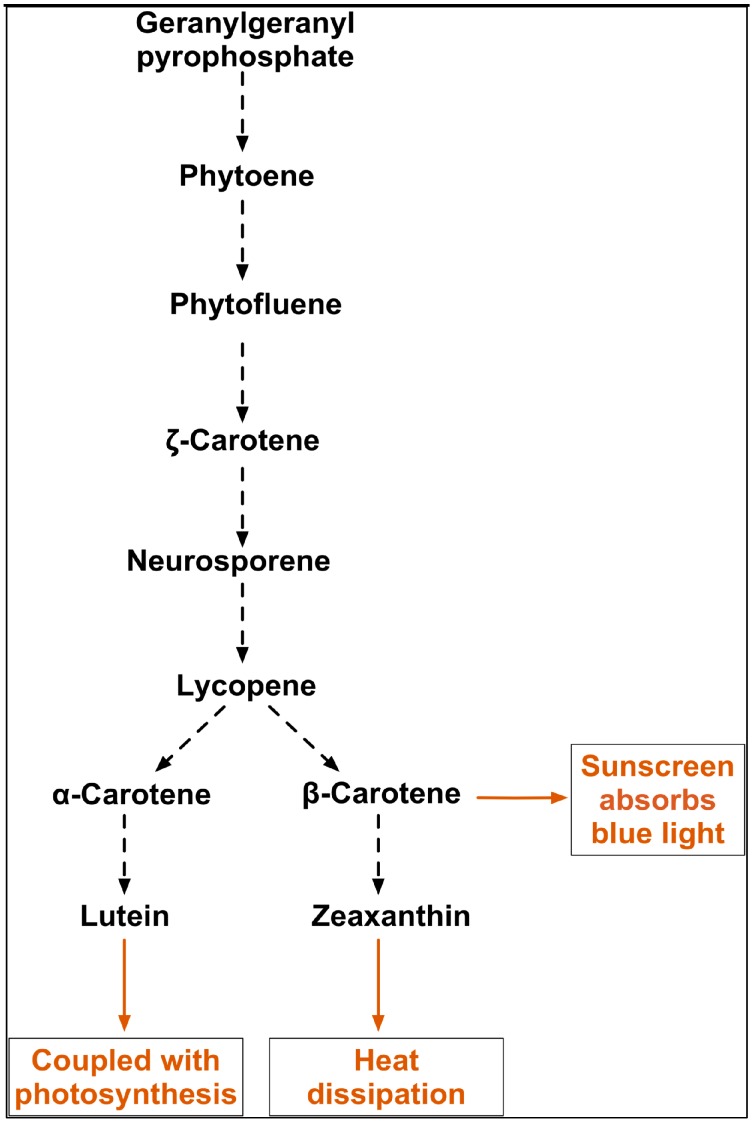
Carotenoids pathway showing synthesis of lutein, β-carotene, α-carotene, and zeaxanthin from phytoene in *D. salina* and possible functions of the major carotenoids (Adapted from [42,43]).

Figure 9 shows the pathway for the synthesis of key carotenoids in this study. Significantly the data presented here show that α-carotene is linearly correlated with β-carotene but not lutein, although α-carotene is the precursor of lutein. Lutein, the most abundant xanthophyll in higher plants, is found in the light harvesting complexes in higher plants and also protects against photodamage. Its most important function is thought to be in quenching triplet chlorophyll (^3^Chl^*^) to prevent energy transfer to molecular oxygen and consequent formation of singlet oxygen (^1^O_2_^*^) [34], but it also quenches excited ^1^Chl^*^ (NPQ) to prevent the formation of reactive oxygen species (ROS) under high light [40]. It also contributes to light harvesting, by transferring excitation energy to chlorophyll, and has a structural role associated with the antenna system [34], consequently the changes in antenna size due to photodamage under high light may also affect lutein content. Lutein in *D. salina* has been previously reported as a growth-coupled primary metabolite with a strong correlation with chlorophyll synthesis, but interestingly, not with light [41]. The finding in this study points to additional interactions involved in the synthesis of lutein, which are linked specifically to chlorophyll synthesis. Moreover, the positive correlation between β-carotene and zeaxanthin may suggest a proportional partitioning of β-carotene into the xanthophyll cycle and the β-carotene plastoglobuli, which is consistent with the idea that two complete pathways for β-carotene biosynthesis exist in *D. barwawil*, one in the chloroplast membranes for the biosynthesis of β-carotene and one in the plastoglobuli for the accumulation of β-carotene [29]. It is possible to conclude that *D. salina* strains have evolved coordinated universal photoprotection mechanisms for the maintenance of high efficiency under high light stress by accumulating carotenoids, in particular β-carotene. However, the effectiveness of these mechanisms varies greatly between strains and therefore the potential for β-carotene production varies among strains.

**Figure 10:**
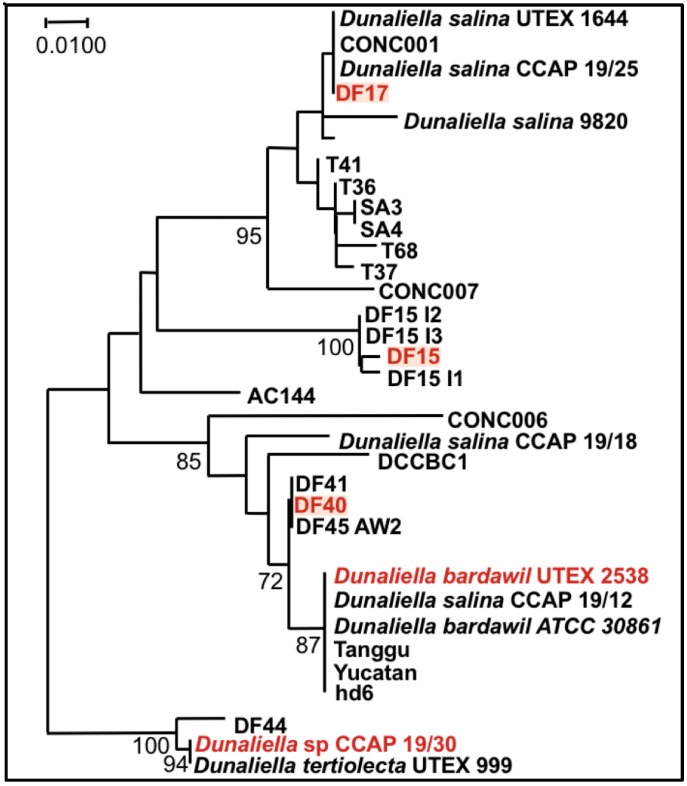
Phylogenetic tree showing the location of the three newly isolated *Dunaliella* strains (DF15, DF17, and DF40) compared to CCAP 19/30 and UTEX 2538 used in this study [44].

Finally, it is noteworthy that the statistical analysis based on the data obtained from the biochemical characterization suggests a grouping of the five strains into three different groups: (1) DF15 and UTEX 2538; (2) DF17 and DF40; and (3) CCAP 19/30 as shown in Figure 8. However, genetic classification using the approaches of bar coding shows a higher similarity between DF40 and UTEX 2538, and therefore groups the five strains into four different groups: (1) DF40 and UTEX 2538, (2) DF17, (3) DF15, and (4) CCAP 19/30 as shown in the phylogenetic tree provided by Dr. Declan Schroeder at The Marine Biological Association, UK [44] (Figure 10). This indicates the complicity of strain classification in *Dunaliella* by using a single classification method, and the importance of strain selection for the commercial production of *Dunaliella* biomass and natural β-carotene.

## 5. Conclusions

This study shows how strain difference plays a significant role in the accumulation of carotenoids in *D. salina*. Carotenoid content increased with the increase of light intensity and contributed to photoprotection against photodamage. Cellular contents of *all-trans* β-carotene, *9-cis* β-carotene, *all-trans* α-carotene and zeaxanthin, but not lutein, were closely correlated with each other, signifying synthesis of these carotenes and zeaxanthin along a metabolic pathway that is under common control. Significantly a strong correlation between lutein and respiration in photosynthetic cells was identified; there was also a strong relationship between lutein, photosynthesis, chlorophyll, and respiration. Among the three newly isolated *D. salina* strains, DF15 produced a significantly higher (>5-fold) content of β-carotene over different light intensities compared to DF17 or DF40, despite the fact that they are all strains of *D. salina*. Physiological study on the biochemical performance of the new isolated strains shows a different grouping strategy to that obtained from genetic classification. The data demonstrate the importance of strain selection from a number of *Dunaliella* strains based on their biochemical performance for the commercial production of β-carotene.

## Figures and Tables

**Table 1:** Two-way ANOVA analysis of the responses of all examined variables (photosynthesis, respiration, doubling time, *all-trans* β-carotene, *9-cis* β-carotene, glycerol, lutein, zeaxanthin, *all-trans* α-carotene, total carotenoids, total chlorophyll) to strain and light intensity and their interaction (Light intensity*Strain). The values of all observations were transformed by taking log function, square root function or reciprocal to fit linear models. Df: degrees of freedom; *F* values: variation between sample means; *P* values: significance levels and a star (*) indicates *P* ≤ 0.05, two stars (**) *P* ≤ 0.01 and three stars (***) *P* ≤ 0.001.

Response	Light Intensity				Strain				Light Intensity*Strain			
	Df	*F* Values	*P* Values	Significance Level	Df	*F* Values	*P* Values	Significance Level	Df	*F* Values	*P* Values	Significance Level
Photosynthesis	3	8.1825	0.0002	***	4	71.2528	<2.2e−16	***	12	2.7966	0.0073	**
Respiration	3	1.7925	0.1641		4	52.7992	1.96e−15	***	12	2.4328	0.0176	*
Total carotenoids	3	2.9403	0.0446	*	4	693.560	<2.2e−16	***	12	7.9749	2.52e−07	***
Total chlorophyll	3	36.529	1.55e−11	***	4	161.782	<2.2e−16	***	12	10.285	8.41e−09	***
*All-trans* β-carotene	3	88.922	<2.2e−16	***	4	474.255	<2.2e−16	***	12	3.6878	0.0009	***
*9-cis* β-carotene	3	28.119	6.02e−10	***	4	730.574	<2.2e−16	***	12	6.8407	1.67e−06	***
Lutein	3	7.3679	0.0005	***	4	118.762	<2.2e−16	***	12	6.4955	3.08e−06	***
Zeaxanthin	3	35.542	2.31e−11	***	4	83.0526	<2.2e−16	***	12	5.2669	3.13e−05	***
*All-trans* α-carotene	3	113.39	<2.2e−16	***	4	408.180	<2.2e−16	***	12	5.9987	7.64e−06	***
Glycerol	3	2.1170	0.1132		4	95.5589	<2.2e−16	***	12	5.0858	4.50e−05	***

(*) indicates *P* ≤ 0.05, (**) *P* ≤ 0.01, (***) *P* ≤ 0.001.
